# Taxon, Arboreality, Sex, and Season—Factors Influencing Gastrointestinal Parasite Occurrence in Zoo‐Housed Non‐Human Primates

**DOI:** 10.1002/zoo.70063

**Published:** 2026-03-26

**Authors:** Stalder Sandro, Hatt Jean‐Michel

**Affiliations:** ^1^ Clinic for Zoo Animals, Exotic Pets and Wildlife, Vetsuisse Faculty University of Zurich Zurich Switzerland

**Keywords:** host species susceptibility, nematodes, protozoa, zoological institution

## Abstract

The emergence of anthelminthic resistance is a well‐documented phenomenon in livestock and companion animals. Given the scarcity of new antiparasitic drugs, the implementation of effective, holistic anthelminthic control strategies is critical to preserving the efficacy of existing treatments. Understanding parasite‐host‐environment interactions is necessary to establish such strategies aiming to reduce the use of anthelminthic drugs, mitigate resistance, and ultimately improve animal health and welfare. This study analyzed 3767 fecal parasitological reports from non‐human primates housed at a Swiss zoological institution over a 48‐year period. Frequency of positive results was analyzed in relation to taxonomy, arboreality, sex, season, and mean monthly temperatures. At least one parasite was detected in 53.2% of the samples, with higher occurrence of protozoa (47.4%) than nematodes (7.8%) and cestodes (0.1%). Due to the large sample number and the extensive study period, it was possible to define taxon‐specific parasite susceptibilities. The effect of arboreality on parasite occurrence was found to be negligible. Seasonal trends were evident, with the highest nematode occurrence in summer, whereas protozoa occurrence peaked in spring and winter. Comprehensive monitoring is a fundamental prerequisite for the development and implementation of targeted and effective deworming strategies. Such strategies extend beyond the regular administration of anthelminthics and include integrated management practices, consideration of environmental factors, and, ideally, ongoing monitoring of resistance patterns. A well‐designed, holistic deworming strategy not only improves animal health and welfare and reduces the risk of zoonotic transmission but also reinforces a zoo's reputation as a center of conservation and education and contributes to the success of reintroduction programs.

## Graphical abstract

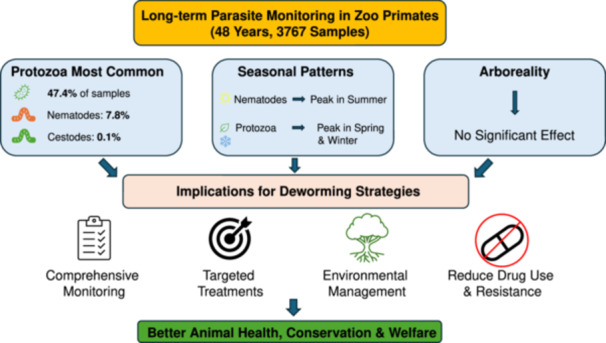

## 1 Introduction

The emergence of anthelminthic resistance is a well‐documented phenomenon in livestock and companion animals, with growing evidence of drug‐resistant helminths also affecting humans (Batista et al. 2024; Charlier et al. 2022; Kaplan and Vidyashankar 2012; Osei‐Atweneboana et al. 2007; Vercruysse et al. 2011). Resistance has been identified across the three major anthelminthic drug classes: Avermectins, benzimidazoles, and cholinergic receptor agonists (Kaplan and Vidyashankar 2012; Beech et al. 2011; Geerts and Gryseels 2001). Given the absence of new antiparasitic drugs over the past two decades and with no new classes of anthelminthic drugs expected to be available in the near future, the implementation of strategic anthelminthic control is critical to preserving the efficacy of existing treatments. Drug‐resistant helminth infections impair animal health, reduce welfare, and represent a significant challenge to disease management. Factors contributing to the development of resistance include high treatment frequency, inappropriate timing, reliance on single‐drug regimens, underdosing, and poorly targeted interventions (Geerts and Gryseels 2001). To mitigate resistance, best practices emphasize targeted therapy, appropriate dosing, strategic timing of treatments, and annual rotation of two effective anthelminthics. In contrast, indiscriminate mass treatments should be avoided, and the use of antiparasitic drugs should be minimized. A targeted, well‐designed treatment regimen is, however, only a part of a holistic parasite control strategy, which also includes comprehensive monitoring and integrated management practices.

In recent years, zoological facilities have adopted various strategies to reduce the use of antiparasitic drugs. These include strict quarantine protocols, interruption of parasite life cycles via removal of feces and intermediate hosts, adjunctive treatments with nonpharmaceutical agents like tannins or copper oxide, and improved management practices such as field rotation (Fontenot and Miller 2012).

Gastrointestinal parasites are a common finding in captive non‐human primates (NHPs), with many species of NHPs harboring zoonotic potential due to their close phylogenetic relationship to humans (Kvapil et al. 2017; Levecke et al. 2007; Li et al. 2015; Pérez Cordón et al. 2008; Soledad Gómez et al. 1996; Vonfeld et al. 2022). Inappropriate antiparasitic treatment regimens in NHPs pose risks not only to animal health but also to human handlers, as resistant parasites may be transmitted zoonotically.

Several host‐related and environmental factors may influence parasite occurrence in captive NHPs. Arboreality may affect exposure risk to infective stages of parasites, as arboreal species may have reduced contact with contaminated substrates compared to ground‐dwelling species. Sex‐based differences in parasitism may arise due to hormonal or behavioral factors influencing immune function or exposure. Taxonomic differences reflect variation in host susceptibility, immunity, or social behavior that may affect parasite transmission. Seasonality may impact parasite life cycles directly via temperature or humidity effects on larval survival or indirectly through changes in host behavior or enclosure conditions.

Routine fecal parasitological testing plays a critical role in identifying clinically significant parasites and guiding targeted, effective, and cost‐efficient anthelminthic regimens. Zoo Zurich has been systematically monitoring endoparasite prevalence in its animal collection for nearly 50 years, providing valuable data on trends in transmission, occurrence, and host susceptibility over time. Building on this extensive dataset, the present study aims to analyze the parasitological fecal examination reports of captive NHPs, to gain a deeper understanding of host‐parasite‐environment interactions. This knowledge is crucial for developing more targeted and cost‐effective parasite prevention strategies that enhance animal welfare while mitigating the risk of zoonotic transmission of resistant endoparasites to animal handlers. This study is distinguished by a long observation period, broad species coverage, and consideration of arboreality and environmental temperatures as factors influencing parasite occurrence. This comprehensive approach provides new insights into parasitic diseases and their management within zoological institutions.

## 2 Materials and Methods

### 2.1 Data

All available results of parasitological fecal examinations in primates carried out between January 1969 and September 2017 were included in this study. These tests were performed as part of routine quarantine screening for newly acquired animals, regular health monitoring of clinically healthy individuals, evaluation of animals exhibiting clinical signs of endoparasitism, or to assess treatment efficacy. However, as the specific reason for testing was not documented in the majority of cases, this variable could not be evaluated further. The parasitological tests were all conducted at the same facility, and results were reviewed by one author (S.S.). Results were reported either qualitatively as “positive” or “negative,” or semi‐quantitatively using symbols; “−” for negative and “+”, “++”, or “+++” to indicate increasing levels of positivity (i.e., weak to strong positive). For the purposes of this study, all positive results, regardless of the number of “+” symbols, were uniformly categorized as “positive.”

The samples were categorized by taxon in old‐world monkeys (OWMs), including all members of the parvorder *Catarrhini*, new‐world monkeys (NWMs), and prosimians, and further by taxonomic family. NHPs were divided into mainly arboreal, mainly ground‐dwelling, and intermediate species based on behavior in the wild to investigate whether this trait influences parasite occurrence (Table 1). Chimpanzees (*Pan troglodytes*) were classified as intermediate as they, especially young animals, frequently travel on arboreal substrate (Sarringhaus et al. 2014).

**Table 1:** Number of fecal samples of non‐human primates analyzed at Zoo Zurich from 1969 to 2017 categorized by taxon.

	Family	Genus/Species	Common name	Arboreality	Samples
Old‐world monkeys					2854
	*Cercopithicedae*				459
		*Cercopithecus sp*.	Vervets and guenons		7
		*Cercopithecus albogularis*	Sykes’ monkey	Arboreal	4
		*Cercopithecus cephus*	Moustached guenon	Arboreal	2
		*Cercopithecus hamlyni*	Hamlyn's monkey	Intermediate	17
		*Cercopithecus nictitans*	Greater spot‐nosed monkey	Arboreal	22
		*Macaca nigra*	Crested black macaque	Intermediate	74
		*Miopithecus sp*.	Talapoins	Arboreal	40
		*Theropithecus gelada*	Gelada	Ground‐dwelling	171
		*Trachypihecus obscurus*	Dusky leaf monkey	Arboreal	122
	*Hominidae*				1658
		*Gorilla gorilla*	Western gorilla	Ground‐dwelling	416
		*Hominidae*	Hominids		4
		*Pan troglodytes*	Chimpanzee	Intermediate	425
		*Pongo abelii*	Sumatran orangutan	Intermediate	813
	*Hylobatidae*				737
		*Hylobates lar*	Lar gibbon	Arboreal	7
		*Hylobates pileatus*	Pileated gibbon	Arboreal	327
		*Symphalangus syndactulus*	Siamang	Arboreal	403
New‐world monkeys					566
	*Atelidae*				75
		*Lagothrix lagotricha*	Common woolly monkey	Arboreal	75
	*Callitrichidae*				368
		*Callimico goeldii*	Goeldi's marmoset	Arboreal	90
		*Callithrix geoffroyi*	White‐headed marmoset	Arboreal	28
		*Callithrix jacchus*	Common marmoset	Arboreal	13
		*Callitrichidae sp*.	Callitrichids	Arboreal	7
		*Cebuella pygmaea*	Western pygmy marmoset	Arboreal	103
		*Leontocebus fuscicollis*	Brown‐mantled tamarin	Arboreal	5
		*Leontopithecus chrysomelas*	Golden‐headed lion tamarin	Arboreal	54
		*Leontopithecus rosalia*	Golden lion tamarin	Arboreal	21
		*Saguinus imperator*	Emperor tamarin	Arboreal	47
	*Cebidae*				83
		*Cebinae*	Capuchin monkeys	Arboreal	65
		*Saimiri sp*.	Squirrel monkey	Arboreal	15
		*Sapajus xanthosternos*	Golden‐bellied capuchin	Arboreal	3
	*Pithecidae*				40
		*Pithecia pithecia*	White‐faced saki	Arboreal	40
Prosimians					361
	*Cheirogaleidae*				44
		*Cheirogaleidae sp*.	Cheirogaleids	Arboreal	2
		*Microcebus lehilahytsara*	Goodman's mouse lemur	Arboreal	42
	*Lemuridae*				290
		*Eulemur albifrons*	White‐headed lemur	Arboreal	3
		*Eulemur mongoz*	Mongoose lemur	Arboreal	54
		*Eulemur rufifrons*	Red‐fronted lemur	Arboreal	6
		*Eulemur rufus*	Red lemur	Arboreal	3
		*Hapalemur sp*.	Bamboo lemur	Arboreal	19
		*Lemur catta*	Ring‐tailed lemur	Intermediate	142
		*Lemuriformes*	Lemuriformes		3
		*Prosimiae*	Prosimians		1
		*Varecia rubra*	Red ruffed lemur	Arboreal	57
		*Varecia variegata*	Black‐and‐white ruffed lemur	Arboreal	2
	*Lorisidae*				27
		*Nycticebus sp*.	Slow lori	Arboreal	27
Total					3781

*Note:* Note that the total sample number differs since 14 mixed samples from different species were attributed to two species in this table.

In addition, samples were categorized by meteorological season at time of sample collection, defined as winter (December–February), spring (March–May), summer (June–August), and autumn (September–November), and, if known, by the sex of the animal.

### 2.2 Statistical Analysis

To evaluate whether parasite occurrence varied significantly among taxonomic groups, meteorological seasons, and sexes, we performed a chi‐square test for independence contingency tables of parasite presence (dependent variable) against taxon group (OWMs, NWMs, and prosimians), meteorological season, and sex (independent grouping variables). Where significant associations were found, pairwise chi‐square tests with Bonferroni correction were conducted to identify specific group differences while controlling for multiple comparisons. Logistic regression analysis was then performed to model the probability of nematode or protozoa presence (dependent variables) as a function of taxon, arboreality, and sex (independent variables). This allowed assessment of individual and combined effects of multiple predictors on parasite occurrence while controlling for potential confounding variables. We fitted a generalized linear model with a binomial response and logit link function, including all main effects and interactions. To interpret the effects of taxon, arboreality, and sex on parasite presence, estimated marginal means (EMMs) were calculated to provide adjustment predictions for each group, accounting for other variables. This approach allowed fair comparisons between groups despite differences in sample sizes or characteristics. Pairwise comparisons of EMMs were adjusted using Tukey's method to control for multiple testing. As all NWM species investigated in this study were classified as mainly arboreal, the same analysis investigating arboreality was conducted including only OWMs to exclude a possible taxonomical confounder. Logistic regression analysis investigating arboreality was also performed for cercopithecids, as this was the only family in which arboreal, intermediate, and ground‐dwelling species were represented. Samples for which enrichment methods to specifically find nematodes (e.g., Baermann funnel) were applied were excluded from the logistic regression model for protozoa. Mixed samples from more than one taxon were excluded from the logistic regression models. Furthermore, to investigate whether changes of temperature within each month over time influences parasite occurrence, mean monthly temperature measured 2 m above the ground at the meteorological station Zurich‐Fluntern (47°22′40.5″ N 8°33′56.7″ E, 558m a.s.l.) were assessed in 5‐year intervals and compared with the occurrence of parasites of the respective time period using Pearson's product moment correlation coefficient and Kendall's tau correlation coefficient (Federal Office of Meteorology and Climatology MeteoSwiss 2024).

All statistical analyses were performed using RStudio 2023.12.1 + 402. A *p*‐value of < 0.05 was considered statistically significant.

## 3 Results

### 3.1 Methods

In total, 3767 parasitological examinations were included covering 32 clearly defined species of 10 families (Table 1). Fourteen samples were mixed samples from more than one species and therefore attributed to two species in Table 1. The parasitological methods performed included enrichment using merthiolate‐iodine‐formaldehyde (MIF) or sodium acetate‐acetic acid‐formalin (SAF) fixative (*n* = 2538), flotation (*n* = 975), cultivation (*n* = 338), Baermann funnel (*n* = 230), Ziehl‐Neelsen staining (*n* = 23), chromotrope base staining (*n* = 21), coproantigen detection (*n* = 5), and sedimentation (*n* = 2). In 534 samples, the applied methods were not stated in the report. These included early samples because until the late seventies, the applied methods were not routinely stated in the reports (Figure S1). From the eighties onwards, enrichment using MIF or SAF was the most frequently used method throughout the remaining timespan. Starting in the early nineties, the MIF or SAF method was routinely combined with a flotation technique. Cultivation was performed commonly in the late seventies until the mid‐eighties, afterwards only sporadically until the year 2000, and has not been performed since. Chromotrope base and Ziehl‐Neelsen staining both started being used in 2001, but whereas chromotrope base staining was only performed in 2001, Ziehl‐Neelsen was sporadically performed in the years after.

### 3.2 Parasites

In 2004, samples (53.2%) at least one parasite was detected. Multiple parasite detection occurred more frequently in OWMs than NWMs and prosimians (Table 2). The highest number of parasite species detected in a single fecal sample was 8 in a gelada baboon (*Theropithecus gelada*).

**Table 2:** Number of samples and number of parasite species detected in old‐world monkeys (OWMs), new‐world monkeys (NWMs), and prosimians at Zoo Zurich between 1969 and 2017.

	0	1	2	3	4	5	6	7	8	Total
OWMs	1098 (38.6)	819 (28.8)	425 (14.9)	256 (9.0)	139 (4.9)	80 (2.8)	25 (0.9)	4 (0.1)	1 (0.04)	2847
NWMs	418 (74.6)	109 (19.5)	24 (4.3)	8 (1.4)	1 (0.2)					560
Prosimians	247 (68.6)	73 (20.3)	22 (6.1)	9 (2.5)	4 (1.1)	5 (1.4)				360

*Note:* Number in brackets represent the percentage of total samples.

In 7.8% of the samples (*n* = 294), nematodes were detected, including *Strongyloides* (*n* = 69), *Enterobius* (*n* = 57), *Trichuris* (*n* = 41), *Ascaris* (*n* = 40), *Strongylida* (*n* = 31), *Spirurida* (*n* = 28), *Ancylostoma* (*n* = 25), *Capillaria* (*n* = 7), *Oesophagostomum* (*n* = 5), *Trichospirura leptostoma* (*n* = 5), and in 5 cases the nematode species was not further classified.

Protozoa were detected in 47.4% of the samples (*n* = 1785). Detected protozoa included *Entamoeba* (*E*.) *coli* (*n* = 1011), *E. histolytica* (*n* = 570), *E. hartmanni* (*n* = 399), *E. chattoni* (*n* = 14), *E. polecki* (*n* = 1), *Balantidium coli* (*n* = 581), *Endolimax nana* (*n* = 387), *Iodamoeba bütschlii* (*n* = 244), *Giardia* (*n* = 144), flagellates (*n* = 129), *Blastocystis* (*n* = 96), *Dientamoeba fragilis* (*n* = 30), *Chilomastix mesnili* (*n* = 12), and coccidia (*n* = 4). Cestodes were detected in two lemurids, two hylobatids, and one cebid and were identified as *Hymenolepis* in all five cases. Detailed percentages of positive samples by parasite and taxa are shown in Table 3.

**Table 3:** Gastrointestinal parasite occurrence rates in non‐human primates at Zoo Zurich from 1969 to 2017 by taxonomic family.

	NWMs	OWMs	Prosimians	Total
*Balantidium coli*	1.8	19.7	2.5	15.4
*Blastocystis*	2.0	2.5	3.6	2.5
*Chilomastix mesnili*	0.4	0.3	0.3	0.3
Coccidia	0.7	N/A	N/A	0.1
*Dientamoeba fragilis*	N/A	0.9	0.8	0.8
*Endolimax nana*	0.5	13.0	3.1	10.2
*Entamoeba chattoni*	N/A	0.5	0.3	0.4
*Entamoeba coli*	3.0	34.2	5.8	26.8
*Entamoeba hartmanni*	0.7	13.5	3.1	10.6
*Entamoeba histolytica*	2.1	19.0	5.3	15.2
*Entamoeba polecki*	N/A	0.0	N/A	0.0
Flagellates	1.6	3.8	3.3	3.4
*Giardia lamblia*	5.0	2.6	12.0	3.8
*Iodamoeba bütschlii*	0.4	8.3	1.9	6.5
*Ancylostoma*	0.9	0.6	0.6	0.7
*Ascaris*	N/A	1.4	N/A	1.1
*Capillaria*	0.4	0.1	0.3	0.2
*Enterobius*	1.4	1.2	4.2	1.5
*Strongylida*	1.8	0.5	1.7	0.8
Unclassified nematodes	N/A	0.2	0.3	0.2
*Oesophagostomum*	N/A	0.2	N/A	0.1
*Spirurida*	4.6	0.0	0.3	0.7
*Strongyloides*	3.6	1.5	1.4	1.8
*Trichospirura leptostoma*	0.9	N/A	N/A	0.1
*Trichuris*	0.4	1.4	N/A	1.1

*Note:* N/A: The parasite was not detected in this group.

Abbreviations: NWMs, new‐world monkeys; OWMs, old‐world monkeys.

### 3.3 Old‐World Monkeys, New‐World Monkeys, Prosimians

Nematodes were significantly more often detected in NWMs (12.9%), compared to OWMs (6.8%). Occurrence rate in prosimians (8.3%) did not differ significantly from OWMs or NWMs. Regarding protozoa, the occurrence rate was significantly higher in OWMs with 56.9% compared to prosimians (25.4%) and NWMs (14.1%). Occurrence rate was also significantly higher in prosimians compared to NWMs. Detailed parasite occurrence by taxa is shown in Table 4.

**Table 4:** Percentage of fecal samples in which parasites were detected by microscopical evaluation using enrichment methods between 1969 and 2017 at Zoo Zurich by taxonomic family.

	Family	Nematodes	Protozoa
OWM	*Cercopithecidae*	11.8^b^	66.2^c^
OWM	*Hominidae*	6.8^b^	58.4^e^
OWM	*Hylobatidae*	3.6^a^	47.6^c^
NWM	*Callitrichidae*	10.8^b^	10.8^ad^
NWM	*Cebidae*	20.5^b^	13.3^abde^
NWM	*Atelidae*	21.3^b^	18.7^abde^
NWM	*Pithecidae*	0^ab^	37.5^bce^
Prosimian	*Cheirogaleidae*	2.3^ab^	9.1^abde^
Prosimian	*Lemuridae*	7.3^ab^	28.5^abd^
Prosimian	*Lorisidae*	25.9^b^	3.7^abde^

*Note:* Letters indicate whether the percentages differ significantly within a column based on a comparison of estimated marginal means from a logistic model accounting for sex, taxon, and behavior. Percentages attributed identical letters do not differ significantly (*p* ≥ 0.05).

Abbreviations: NWM, new‐world monkey; OWM, old‐world monkey.

### 3.4 Arboreality

When including all taxa, the occurrence rate of nematodes was significantly higher in ground‐dwelling (10.7%) compared to intermediate (5.9%) species. Occurrence in arboreal species (8.5%) did not differ significantly from the other groups.

Regarding protozoa, occurrence rates of all three groups differed significantly from each other based on the logistic model. Occurrence was highest in intermediate species (67.2%), followed by ground‐dwelling (66.9%) and arboreal (38.5%) species (Table 5).

**Table 5:** Percentage of fecal samples of non‐human primates in which parasites were detected by microscopical evaluation using enrichment methods between 1969 and 2017 at Zoo Zurich by arboreality.

	Nematodes	Protozoa
Arboreal	8.5^ab^	38.5^a^
Intermediate	5.9^b^	67.2^b^
Ground‐dwelling	10.7^a^	66.9^c^

*Note:* Letters indicate whether percentages differ significantly within a column based on a comparison of estimated marginal means from a logistic model accounting for sex, taxon, and behavior. Percentages attributed identical letters do not differ significantly (*p* ≥ 0.05).

When the analysis was conducted separately for OWMs and cercopithecids, no significant difference in the occurrence of protozoa or nematodes was observed between arboreal and ground‐dwelling species in either group (Tables 6 and 7).

**Table 6:** Percentage of fecal samples of old‐world monkeys in which parasites were detected by microscopical evaluation using enrichment methods between 1969 and 2017 at Zoo Zurich by arboreality.

	Nematodes	Protozoa
Arboreal	5.7^ab^	59.8
Intermediate	5.9^a^	69.3
Ground‐dwelling	10.7^b^	66.9

*Note:* Letters indicate whether percentages differ significantly within a column based on a comparison of estimated marginal means from a logistic model accounting for sex, taxon, and behavior. Percentages attributed identical letters do not differ significantly (*p* ≥ 0.05).

**Table 7:** Percentage of fecal samples of cercopithecids in which parasites were detected by microscopical evaluation using enrichment methods between 1969 and 2017 at Zoo Zurich by arboreality.

	Nematodes	Protozoa
Arboreal	13.7^a^	69.5
Intermediate	3.3^b^	72.7
Ground‐dwelling	14.6^a^	79.6

*Note:* Letters indicate whether percentages differ significantly within a column based on a comparison of estimated marginal means from a logistic model accounting for sex and behavior. Percentages attributed identical letters do not differ significantly (*p* ≥ 0.05).

### 3.5 Sex

No significant difference in protozoa occurrence in males and females was observed. Nematodes were significantly more often detected in males (7.9%) than in females (4.8%).

### 3.6 Season and Climate

**Figure 1:**
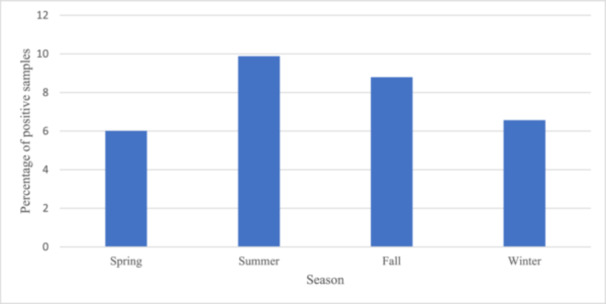
Percentage of fecal samples of non‐human primates in which nematodes were detected by microscopical evaluation using enrichment methods between 1969 and 2017 at Zoo Zurich by season.

In nematodes, a seasonal trend was observed. Nematode occurrence peaked in summer with 9.9% positive samples and decreased in fall (8.8%) and winter (6.6%) with the lowest occurrence in spring (6.0%) (Figure 1). Nematode occurrence was significantly higher in summer compared to spring.

**Figure 2:**
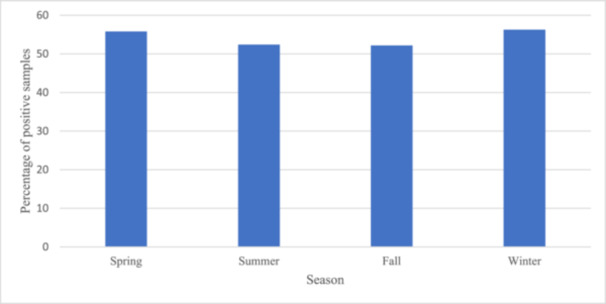
Percentage of fecal samples of non‐human primates in which protozoa were detected by microscopical evaluation using enrichment methods between 1969 and 2017 at Zoo Zurich.

Protozoa occurrence was highest in winter (56.3%), followed by spring (55.8%), summer (52.4%), and fall (52.2%). A trend towards higher percentages of positive samples in colder months was observed, although not statistically significant (Figure 2).

**Figure 3:**
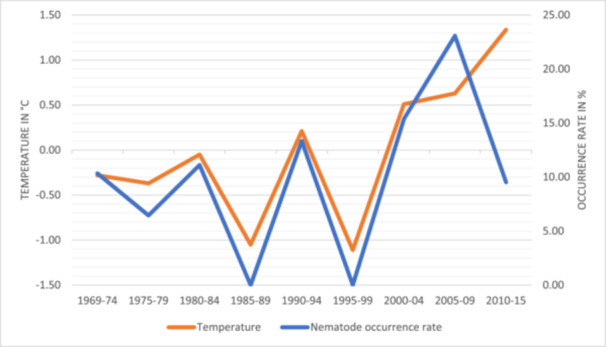
Monthly average temperature in the month of November, measured by a meteorological station in Zurich, and mean occurrence rate of nematodes in fecal samples of non‐human primates at Zoo Zurich in 5‐year intervals. (Temperature: Difference between the overall average temperature in November throughout the study period and the average temperature in November measured in the respective 5‐year period, for example, 2010–2015: The average temperature in November during 2010–2015 was 1.34°C above the average monthly temperatures in November from 1969 to 2015).

When comparing the mean monthly temperature with nematode occurrence, a statistically significant positive correlation was observed for the month of November, where the percentage of positive samples increased with increased mean temperature (Figure 3) (Pearson correlation coefficient = 0.73, Kendall's Tau = 0.70, *p* = 0.025). No statistically significant correlation was observed for any other month.

No significant correlation between protozoa occurrence and monthly temperature was observed in any month throughout the study period.

## 4 Discussion

In this study, the results of fecal parasitological examinations of NHPs at the Zoo Zurich from 1969 to 2017 were analyzed. In 53.2% of the 3767 samples included, nematodes or protozoa were detected. Previous studies reported occurrence rates of 22% to 100% (Kvapil et al. 2017; Levecke et al. 2007; Pérez Cordón et al. 2008; Vonfeld et al. 2022; Köster et al. 2022; Fagiolini et al. 2010). This wide range might be attributed to differences in investigated species, diagnostic methods, quarantine protocols, sampling, and deworming strategies between the investigated institutions. The overall occurrence of protozoa was higher (47.4%) compared to that of nematodes (7.8%), which is in agreement with previous reports (Levecke et al. 2007; Pérez Cordón et al. 2008; Vonfeld et al. 2022). There are, however, studies reporting higher occurrence rates of nematodes (Kvapil et al. 2017; Fagiolini et al. 2010; Aviruppola et al. 2016; Martin‐Solano et al. 2017). The findings of this study suggest that nematodes are more often found in NWMs than in OWMs and protozoa vice versa. This could be one reason for higher nematode occurrence found in studies where mainly or only NWMs were investigated (Kvapil et al. 2017; Martin‐Solano et al. 2017). Another important factor influencing the occurrence rate is the diagnostic method. While flotation or combined sedimentation‐flotation is routinely used for helminth detection, several methods are commonly used to detect protozoa, differing in sensitivity (Marti and Escher 1990; Fitri et al. 2022). Therefore, reported occurrence rates should be compared with caution when different methods for parasite detection were applied.

*Strongyloides* were the most frequently detected nematodes in this study, with the highest occurrence in lorisids (14.8%) and cebids (13.3%). Three species of *Strongyloides* are of importance in captive NHPs, namely *S. stercoralis* and *S. fuelleborni* in OWMs and *S. cebus* in NWMs. While the first two have a zoonotic potential, *S. cebus* has so far only been detected in NWMs and ring‐tailed lemurs (*Lemur catta*) (Mati et al. 2013; Hiebert et al. 2023). Infection with cryptic *Strongyloides* has been reported in wild Bornean slow loris (Frias et al. 2018). It would be reasonable to assume that soil‐transmitted parasites like *Strongyloides* are more prevalent in ground‐dwelling NHPs, however, this was not the case in this study. Microscopic identification of *Strongyloides* can be complicated by the presence of free‐living nematodes, which may contaminate the sample. As this contamination occurs rapidly and immediate sample collection is not always practicable in a zoological institution, false positive results are possible (Viney and Lok 2018). Furthermore, the trend towards more naturalistic husbandry, including soil as substrate, may lead to an increase of free‐living nematodes in the ground, therefore making rapid sample collection even more important to achieve a correct diagnosis. Effective treatment protocols using ivermectin have been described in both NHPs and humans (Mati et al. 2013; Buonfrate et al. 2022). To prevent the introduction of *Strongyloides* into a captive collection of NHPs, all newly arriving animals should be tested during quarantine using Baermann larvoscopy or Koga agar plate culture (Nosková et al. 2024).

Nematodes mainly or only found in NWM in this study included *Spirurida* and *Trichospirura leptostoma*, respectively, belonging to the order *Spirurida*. These nematodes were mainly detected in callitrichids, with *Spirurida* being detected in 7.2% and *Trichospirura leptostoma* in 1.4% of the samples. It is possible that most, if not all *Spirurida* reported were *Trichospirura leptostoma*, as the *Spirurida* detected were not further classified. *Trichospirura leptostoma* has been associated with a marmoset wasting disease‐like syndrome in common marmosets (*Callithrix jacchus*) (Pfister et al. 1990; Beglinger et al. 1988). Cockroaches are the intermediate host and necessary for the development of *Trichospirura leptostoma* (Beglinger et al. 1988). This should be considered when implementing a feeding and pest control strategy for marmoset species.

The occurrence rate of protozoa in OWMs was higher than in NWMs, which is in agreement with previous reports (Kvapil et al. 2017; Levecke et al. 2007; Vonfeld et al. 2022). One common explanation for this finding is that OWMs are generally more ground‐dwelling and therefore in more contact with contaminated soil than the more arboreal NWM species. A tendency for arboreal NHPs species to be less affected by gastrointestinal parasites has been discussed before (Li et al. 2015; Martin‐Solano et al. 2017; Munene et al. 1998). In the present study, differences of parasite occurrence between arboreal and ground‐dwelling species were investigated when including OWM and NWM species, only OWM species, and species of the cercopithecid family only. Parasite occurrence rates were higher in ground‐dwelling species for protozoa and nematodes in all three cases. When all taxa were included in the logistic model, protozoa occurrence differed significantly between ground‐dwelling and arboreal species, supporting the theory that this behavior does affect the occurrence of protozoa. When only OWMs or cercopithecids were included, the difference was no longer statistically significant. This might be attributed to a smaller number of samples or the fact that fewer species were included, increasing the relevance of other factors (e.g., taxon). However, the finding that protozoa occurred in 59.8% of samples in arboreal OWMs and only in 14.1% of arboreal NWMs suggests that other factors than arboreality are more relevant in influencing the occurrence of protozoa (e.g., taxon‐specific susceptibility). Regarding nematodes, occurrence did not differ significantly between arboreal and ground‐dwelling species in any logistic model, suggesting that arboreality does not affect nematode occurrence in captive NHPs.

Studies on the seasonality of gastrointestinal parasitosis have been conducted in wild NHPs with varying results (Yang et al. 2022; Rondón et al. 2017; Blersch et al. 2021). In the present study, a trend towards higher nematode occurrence in summer and higher protozoa occurrence in winter was found. As numerous NHP species at Zoo Zurich are housed indoors year‐round (lemurs, callitrichids) or temporarily during cold seasons, the influence of seasonality and climate may be reduced. It is known that climate change influences parasitic disease occurrence in several habitats (Short et al. 2017). A statistically significant positive correlation between average monthly temperature and nematode occurrence was evident for the month of November in the current study. A clear rise in temperature is evident from the year 2000 onwards, which may be explained by global warming. Although parasite occurrence rises along with the temperature in the first 10 years, the curves are drifting apart in the years 2010–2015, with the temperature increasing further but a decrease in parasite occurrence (Figure 3). This decoupling of correlation might be explained by changes of testing or treating regimens, animal housing and management, or changes of the species present at the facility in these years. No correlation was evident in any other month. However, there are several factors that could have decisively altered this analysis (e.g., indoor housing, antiparasitic treatment regime, number of samples), and further investigations are necessary to determine whether and how rising temperatures and changing climate affect animals kept in zoological institutions, particularly those housed outdoors.

One limitation of this study is that, primarily in early samples, the applied enrichment method was not routinely stated in the parasitological report. Therefore, it is unknown whether parasites emerging at later timepoints were absent or not diagnosed due to inadequate testing procedures.

This study assessed fecal parasitological results of captive NHPs in a Swiss zoological institution over nearly 50 years. The results of this study showed that gastrointestinal parasites were highly prevalent in OWMs throughout the study period, including potentially zoonotic pathogens like *Entamoeba histolytica*, *Strongyloides,* and *Trichuris*. Several NHP families' particular susceptibilities towards specific parasite infestation were demonstrated, for example, *Entamoeba* in OWMs, *Spirurida* in callitrichids, *Giardia* in lemurs, and *Ascaris* in hominids. While nematodes were present, protozoa appear to pose the greater challenge in terms of parasite control. This emphasizes the importance of careful drug management, especially since the selection of available treatments for protozoa is even more limited than for nematodes. Cestodes appeared to be a minor issue in the current study, likely due to the absence of intermediate hosts necessary for completing their life cycle. The effect of arboreality on the occurrence of gastrointestinal parasites was shown to be negligible, and arboreal behavior should not be a justification to reduce preventative measures. Endoparasites were found year‐round, with a trend towards higher occurrence of nematodes in summer and higher occurrence of protozoa in colder months. To better understand the influence of seasonality on endoparasite loads and its implications for prevention strategies, further research is needed, particularly in species housed outdoors year‐round. Additionally, the impact of climate change on the occurrence and distribution of endoparasites in animals under human care remains largely unexplored and warrants further investigation.

Comprehensive monitoring is a fundamental prerequisite for the development and implementation of targeted and effective deworming strategies. Such strategies extend beyond the regular administration of anthelminthics and include standardized hygiene and quarantine protocols, integrated management practices, consideration of environmental factors, and, ideally, ongoing monitoring of resistance patterns. Integrated management practices aim to optimize all aspects of an animal's environment and husbandry to minimize infection risk and interrupt transmission pathways within a facility. Examples are the development of effective strategies to control intermediate hosts, implementation of routine testing of caretaker's fecal samples to prevent reverse zoonotic transmission or addressing structural components such as flooring type. Previous studies suggest that flooring type significantly impacts parasite prevalence, with higher burdens observed in animals housed on concrete floors compared to those kept on natural substrates (Mapagha‐Boundoukou et al. 2024; Opeyemi et al. 2022). Over time, many NHP enclosures at Zoo Zurich were converted into more naturalistic exhibits, including a shift from concrete floors to natural substrates. While analysis of this factor was beyond the scope of this study, it represents a promising area for future research. A well‐designed, holistic parasite control strategy not only improves animal health and welfare but also reduces the risk of zoonotic transmission. Furthermore, it reinforces a zoo's reputation as a center of conservation and education and contributes to the success of reintroduction programs, where healthy animals are crucial for successful outcomes (Moreno Mañas et al. 2019).

## Ethics Statement

The authors have nothing to report.

## Supporting information


**Figure 1:** Number of samples and enrichment methods used for microscopical examination of fecal samples of non‐human primates at Zoo Zurich from 1969 to 2017MIF: Fixation using merthiolate‐iodine‐formaldehyde, SAF: Fixation using sodium acetate‐acetic acid‐formalin.

## Data Availability

The data that support the findings of this study are available from the corresponding author upon reasonable request.
