# Supplementary material for: Taxon, Arboreality, Sex, and Season—Factors Influencing Gastrointestinal Parasite Occurrence in Zoo‐Housed Non‐Human Primates
Source: Zoo Biol. 2026 Mar 26;45(4):371–81. doi: 10.1002/zoo.70063 (PMC13446432; doi:10.1002/zoo.70063)
Supplement: Supplementary file 1 [file ZOO-45-371-s001.docx]

Number of samples

| Year | Flotation | MIF/SAF | Method not stated | Cultivation | Baermann funnel | Chromotrope base stain | Coproantigen detection | Ziehl-Neelsen stain | Sedimentation |
| --- | --- | --- | --- | --- | --- | --- | --- | --- | --- |
| 1969 |  |  | 71 |  |  |  |  |  |  |
| 1970 |  |  | 88 |  |  |  |  |  |  |
| 1971 | 19 | 15 | 124 |  |  |  |  |  |  |
| 1972 | 72 | 18 | 1 |  |  |  |  |  |  |
| 1973 | 28 | 3 | 32 | 1 | 1 |  |  |  |  |
| 1974 |  | 4 | 42 | 2 |  |  |  |  |  |
| 1975 | 1 | 99 | 119 |  |  |  |  |  |  |
| 1976 |  | 119 | 58 | 11 |  |  |  |  |  |
| 1977 |  | 77 | 5 | 12 |  |  |  |  |  |
| 1978 | 1 | 75 | 2 |  |  |  |  |  |  |
| 1979 |  | 54 | 8 | 38 |  |  |  |  |  |
| 1980 |  | 85 | 1 |  |  |  |  |  |  |
| 1981 | 3 | 98 | 1 |  | 1 |  |  |  | 1 |
| 1982 | 11 | 143 |  |  | 50 |  |  |  |  |
| 1983 | 10 | 85 |  | 49 | 67 |  |  |  |  |
| 1984 |  | 144 |  | 125 |  |  |  |  |  |
| 1985 | 2 | 251 |  | 8 |  |  |  |  |  |
| 1986 | 2 | 110 |  | 51 |  |  |  |  |  |
| 1987 | 2 | 90 |  | 5 | 3 |  |  |  |  |
| 1988 |  | 101 |  | 5 |  |  |  |  |  |
| 1989 | 1 | 60 |  | 3 | 1 |  |  |  |  |
| 1990 | 1 | 56 |  | 12 |  |  | 1 |  |  |
| 1991 | 2 | 33 |  | 6 |  |  |  |  |  |
| 1992 | 13 | 31 |  |  |  |  |  |  |  |
| 1993 | 29 | 28 |  | 10 |  |  |  |  |  |
| 1994 | 37 | 42 |  | 4 |  |  |  |  |  |
| 1995 | 22 | 24 |  | 1 | 16 |  |  |  |  |
| 1996 | 16 | 18 |  |  | 14 |  |  |  |  |
| 1997 | 16 | 17 |  | 3 | 6 |  |  |  |  |
| 1998 | 21 | 28 |  | 8 | 5 |  |  |  |  |
| 1999 | 5 | 9 |  |  |  |  |  |  |  |
| 2000 | 6 | 16 |  | 8 | 8 |  |  |  |  |
| 2001 | 35 | 47 |  |  | 13 | 21 |  | 11 |  |
| 2002 | 28 | 30 |  |  | 1 |  |  |  |  |
| 2003 | 25 | 25 |  |  | 1 |  |  |  |  |
| 2004 | 36 | 31 |  |  | 7 |  |  | 1 |  |
| 2005 | 60 | 60 |  |  | 3 |  |  | 1 |  |
| 2006 | 60 | 55 |  |  | 8 |  | 1 | 2 |  |
| 2007 | 45 | 46 |  |  | 5 |  |  |  | 1 |
| 2008 | 43 | 40 |  |  | 1 |  |  |  |  |
| 2009 | 38 | 34 |  |  | 2 |  | 1 | 1 |  |
| 2010 | 48 | 45 |  |  | 4 |  | 1 | 1 |  |
| 2011 | 34 | 33 |  |  | 5 |  |  |  |  |
| 2012 | 40 | 39 |  |  |  |  | 1 |  |  |
| 2013 | 47 | 46 |  |  | 3 |  |  | 2 |  |
| 2014 | 42 | 38 |  |  | 1 |  |  | 1 |  |
| 2015 | 33 | 33 | 1 |  | 1 |  |  |  |  |
| 2016 | 37 | 34 |  |  | 1 |  |  | 3 |  |
| 2017 | 1 | 1 |  |  | 2 |  |  |  |  |

Supplementary Fig. 1. Number of samples and enrichment methods used for microscopical examination of fecal samples of non-human primates at Zoo Zurich from 1969 to 2017
MIF: Fixation using merthiolate-iodine-formaldehyde, SAF: Fixation using sodium acetate-acetic acid-formalin
